# The Only African Wild Tobacco, *Nicotiana africana*: Alkaloid Content and the Effect of Herbivory

**DOI:** 10.1371/journal.pone.0102661

**Published:** 2014-07-15

**Authors:** Danica Marlin, Susan W. Nicolson, Abdullahi A. Yusuf, Philip C. Stevenson, Heino M. Heyman, Kerstin Krüger

**Affiliations:** 1 Department of Zoology and Entomology, University of Pretoria, Private Bag X20, Pretoria, South Africa; 2 Royal Botanic Gardens, Kew, Surrey, United Kingdom; 3 Natural Resources Institute, University of Greenwich, Chatham, Kent, United Kingdom; 4 Department of Plant Sciences, University of Pretoria, Private Bag X20, Pretoria, South Africa; Natural Resources Canada, Canada

## Abstract

Herbivory in some *Nicotiana* species is known to induce alkaloid production. This study examined herbivore-induced defenses in the nornicotine-rich African tobacco *N. africana*, the only *Nicotiana* species indigenous to Africa. We tested the predictions that: 1) *N. africana* will have high constitutive levels of leaf, flower and nectar alkaloids; 2) leaf herbivory by the African bollworm *Helicoverpa armigera* will induce increased alkaloid levels in leaves, flowers and nectar; and 3) increased alkaloid concentrations in herbivore-damaged plants will negatively affect larval growth. We grew *N. africana* in large pots in a greenhouse and exposed flowering plants to densities of one, three and six fourth-instar larvae of *H. armigera,* for four days. Leaves, flowers and nectar were analyzed for nicotine, nornicotine and anabasine. The principal leaf alkaloid was nornicotine (mean: 28 µg/g dry mass) followed by anabasine (4.9 µg/g) and nicotine (0.6 µg/g). Nornicotine was found in low quantities in the flowers, but no nicotine or anabasine were recorded. The nectar contained none of the alkaloids measured. Larval growth was reduced when leaves of flowering plants were exposed to six larvae. As predicted by the optimal defense theory, herbivory had a localized effect and caused an increase in nornicotine concentrations in both undamaged top leaves of herbivore damaged plants and herbivore damaged leaves exposed to one and three larvae. The nicotine concentration increased in damaged compared to undamaged middle leaves. The nornicotine concentration was lower in damaged leaves of plants exposed to six compared to three larvae, suggesting that *N. africana* rather invests in new growth as opposed to protecting older leaves under severe attack. The results indicate that the nornicotine-rich *N. africana* will be unattractive to herbivores and more so when damaged, but that potential pollinators will be unaffected because the nectar remains alkaloid-free even after herbivory.

## Introduction

Plants have evolved a variety of chemical defenses to fend off a multitude of attackers [1]. Plants may use both constitutive and herbivore-induced defenses; these can involve primary as well as secondary metabolism, as in compensatory growth that occurs as a plastic phenotypic response to damage [2], [3]. Due to the cost of producing defensive chemicals [4], [5] plants may synthesize these chemicals only after damage has occurred [6]. Whilst constitutive defenses may be costly, a disadvantage of induced defenses is that there is a time delay between herbivore damage and a plant’s response to the damage [7], (but see [8]), essentially leaving the plant susceptible to attack before the induced defenses are activated. Based on the costs of a defense, the value of a plant part and the probability of that plant part being attacked by herbivores, the optimal-defense theory predicts that plant parts that are most valuable and most likely to be attacked will be allocated greatest defenses [9], [10], i.e. reproductive organs and organs necessary for survival [11]. Furthermore, induced defenses are also costly: when the roots of *Nicotiana attenuata* (Solanaceae) plants were treated with methyl jasmonate (a hormone induced by wounding), the induced plants produced 26% fewer seeds than non-induced controls [12], indicating that induced defenses can be detrimental to plant reproduction. Fitness reductions associated with herbivore resistance are common in plants (for reviews see [13], [14]).

Species of *Nicotiana* L. have been used as model systems to study the role of secondary metabolites in plant-herbivore and plant-pollinator relationships [15], [16], [17]. These studies have mostly concentrated on *Nicotiana* species with nicotine as the principal alkaloid. The four main alkaloids found in the genus *Nicotiana* are nicotine, nornicotine, anabasine and anatabine [18] with nicotine occurring in almost all species [19]. Nicotine is highly toxic to many insects [8], [20], but nornicotine and anabasine are known to be even more toxic than nicotine [21], [22].

Leaf damage in *Nicotiana* species has been shown to induce higher alkaloid levels in the vegetative tissues [23], [24], [25] and also in the flowers and nectar [17], [26], [27]. For example, leaf damage increases the nicotine concentration of *N. attenuata*, both locally in damaged leaves and systemically throughout the plant [16]. In addition, increased alkaloid concentrations in the corollas of *N. attenuata* [28] lead to reduced herbivory of the flowers and fruits [29]. Whilst leaf damage can double the nicotine accumulation rate of *N. attenuata*, stalk removal can quadruple the rate [16]. In nornicotine-rich *N. repanda* and *N. trigonophylla*, and nicotine-rich *N. glauca*, leaf damage causes up to a five-fold increase in the principal leaf alkaloid pools [16].

Nectar nicotine concentrations in species such as *N. attenuata* are highly variable between and among populations, and even between flowers in the same inflorescence [30]. These authors showed that the rate of outcrossing, which provides genetic variability, increases when nectar nicotine increases because pollinators move between flowers more frequently in search for low-nicotine containing nectar. Adler et al. [31] compared the pollinator reliance and alkaloid levels of 32 *Nicotiana* species, excluding *N. africana*, and found that selfing species have significantly higher plant tissue nicotine concentrations than outcrossing species.

The genus *Nicotiana* contains 86 recognized species [32], [33], [34], with approximately 75% occurring naturally in the Americas and 25% in Australia. It is likely that the genus evolved east of the Andes in southern South America and dispersed from there to south-western North America, Australia and Africa [35]. The only *Nicotiana* species indigenous to Africa is *N. africana.* This species is endemic to Namibia where it is known from three isolated populations confined to the Brandberg, Erongo and Spitzkoppe mountains in the north of the country. The plants occur in very arid karroid shrubland in a semi-desert/desert environment, and tend to grow in inaccessible, shady areas between granite boulders [36]. *Nicotiana africana* belongs to the section *Suaveolentes*; species in this section are largely endemic to arid regions of continental Australia, with four species occurring on Pacific islands [37]. The defenses of this species have never been examined and nothing is known about its herbivores or pollinators. Saitoh et al. [19] and Sisson and Severson [38] measured the constitutive alkaloid content of *N. africana* leaves and showed that this species accumulates nornicotine as its principal leaf alkaloid.

This is the first study to examine herbivore-induced defenses in *N. africana*. The main motivation was the paucity of data on the effect of herbivory on nornicotine-rich in contrast to nicotine-rich *Nicotiana* species. To the best of our knowledge, the only other study on nornicotine-dominated species is that by Baldwin and Ohnmeiss [16]; in that study leaves were damaged mechanically and not by herbivores. Our aims were to determine the concentrations of the three alkaloids nicotine, nornicotine and anabasine in the leaves, flowers and nectar of *N. africana* under greenhouse conditions, and to examine whether leaf herbivory by the African bollworm, *Helicoverpa armigera* (Lepidoptera: Noctuidae), causes an increase in concentrations of these alkaloids. *Helicoverpa armigera* was chosen as the experimental herbivore because it is indigenous to Africa and occurs in Namibia [39]. This polyphagous species has been recorded feeding on leaves of *N. tabacum* [40] and is therefore likely to adapt to *N. africana*. The following predictions were tested: 1) *N. africana* will have high constitutive levels of leaf, flower and nectar alkaloids; 2) leaf herbivory by *H. armigera* will induce increased alkaloid levels in leaves, flowers and nectar; and 3) increased alkaloid levels will have a negative impact on larval growth.

## Materials and Methods

### Ethics statement

No specific permits were required for the field collection of *Helicoverpa armigera*. Verbal approval for insect sampling was obtained from private landowners. The study did not involve endangered or protected species.

### Plant

Seeds of *N. africana* were obtained from the Institute of Soil Science and Plant Cultivation, State Research Institute, Pulawy, Poland. Seeds were sown in pots (15 cm×15 cm, and 15 cm high) in a soil mixture of 4∶1 river sand and coco peat. The seedlings were kept in a constant environmental chamber at approximately 25°C, natural humidity and 16∶8 h L:D photoperiod. Once the plants reached growth stage 16 with six leaves unfolded (BBCH scale) [41], [42] and these leaves were longer than 4 cm, they were transferred to a greenhouse and transplanted to large pots (38 cm diameter, 43 cm high). Pots of this size were used to avoid pot-bound plants because some *Nicotiana* species, e.g. *N. sylvestris*, cannot be induced to increase their alkaloid content if they are pot-bound [23]. Plants in the greenhouse were maintained in natural light conditions under 50% shade netting, watered as needed and fertilized with MultiFeed Classic (NPK 19∶8∶16) every 14 days. The experiments took place during the late (February, March) and early (October, November) summer months of 2012 when larvae were available. Experiments were carried out at 27.53±1.25°C (mean ± SE) and a relative humidity (RH) of 48±4% RH, with average maximum and minimum temperatures of 36.43±0.74°C and 18.64±0.41°C, and a maximum and minimum of 74±2% and 22±1% RH. Plants were exposed to larvae when they had reached BBCH scales [41], [42] of between 55 (inflorescence emerged, first corolla visible but still closed) and 65 (50% of flowers opened).

### Insect

*Helicoverpa armigera* has six larval stages, and the whole life cycle can be completed in a month under optimum conditions. Eggs of *H. armigera* were obtained from a culture maintained at the Agricultural Research Council - Grain Crops Institute, Potchefstroom, South Africa. The culture was established with specimens collected from light traps during September and October 2011 in Potchefstroom, North-West province (26°43′48.83″S, 27°4′47.53″E). The fifth cultured generation or older was used for experiments. Larvae were reared singly on fresh *N. africana* leaf material inside Petri dishes (9 cm diameter). The Petri dishes were kept in an incubator at 25°C, 50–70% RH and 16∶8 h L:D photoperiod. Larvae were used for experiments when they had reached the fourth instar. They were weighed immediately before and after being used in experiments.

### Herbivory treatments and sampling of plant material

Experiments were carried out in a greenhouse at the University of Pretoria. Efforts to grow *N. africana* plants in the field at different localities in the Pretoria region proved fruitless.

**Figure 1:**
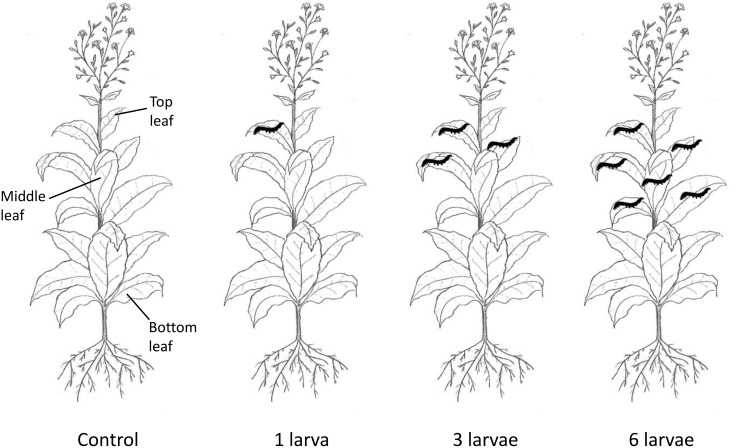
Experimental design. Diagram of *Nicotiana africana* showing the leaves onto which fourth-instar larvae of *Helicoverpa armigera* were placed for experimental purposes. Drawing by F. Venter.

We randomly assigned plants to the following treatments: 1) control (no larvae), 2) one larva per plant, 3) three larvae per plant, and 4) six larvae per plant (Figure 1). The treatments were positioned in a randomized block design and each treatment was replicated five times. Larvae of *H. armigera* are cannibalistic and were therefore placed singly on individual leaves starting from the second-youngest expanded leaf and on successively older leaves when more than one larva was used in the treatment. The “top” expanded leaf was always undamaged and each plant had an undamaged “middle” and “bottom” leaf, except plants that had six larvae on them since on these plants the “middle” leaf would be damaged by larval feeding (Figure 1). All leaves with larvae were enclosed in gauze bags to prevent the larvae from escaping. Leaves on control plants were also enclosed in gauze bags to maintain the same experimental conditions. Larvae were allowed to feed freely on their allocated leaves for four days, after which they were removed; none of the larvae died during the four days. The relative growth rate of larvae during the four-day feeding period on *N. africana* plants was determined as (final mass – initial mass)/initial mass. Leaves were photographed before and after herbivory to record larval feeding, and the image analysis software ImageJ 1.45s (Wayne Rasband, National Institutes of Health, USA, http://imagej.nih.gov/ij) was used to determine the amount of leaf area consumed.

Leaves were excised at the base of the petiole using a razor blade six days after larvae were removed. The time was chosen because nicotine levels in other *Nicotiana* species (i.e. *N. attenuata*, *N. sylvestris*) have been found to peak after five days and remain elevated for about ten days after induction [16], [43]. Leaves were harvested in the morning between 08h00 and 10h00. The following leaves were harvested from each plant: all leaves damaged by herbivory, the top leaf (first unfolded leaf more than 4 cm in length), one middle leaf (approximately leaf six counting from the top) and the oldest bottom leaf, unless this had already senesced, in which case the next oldest leaf was chosen. Harvested leaves were weighed and immediately frozen in liquid nitrogen, ground to a fine pulp using a pestle and mortar, and stored at −20°C in glass vials until they could be freeze-dried before alkaloid extraction.

Depending on availability, we collected nine or ten flowers from each of seven plants. Flowers were excised at the peduncle and the floral tissue thus included sepals, petals, and all of the reproductive parts. Flowers from each plant were pooled and thereafter processed in the same manner as the leaves.

Nectar was collected from all flowering plants immediately before the leaves were harvested. This was done by inserting a 75 µl glass microcapillary tube near the base of the corolla; its volume was measured from the column length, and then pooled for each plant. The nectar sugar concentration was measured using a hand-held refractometer (Eclipse 45–81, Bellingham & Stanley Ltd., UK). The nectar samples were also stored at −20°C until they could be freeze-dried before alkaloid extraction.

### Leaf and flower alkaloid extraction

To determine nicotine, nornicotine and anabasine concentrations, leaf and flower material was extracted with 100 mM KH_2_PO_4_ made strongly acidic (pH<1.1) by adding hydrochloric acid. Sufficient KH_2_PO_4_ solution was added to each sample to cover the leaf material in the glass vial, approximately 1∶3 (w/v). Samples were sonicated for 7 min and thereafter centrifuged at 3500 rpm for 10 min. The pellet was discarded and solid phase extraction using Strata X-C columns (Phenomenex) was used according to manufacturer instructions as follows: columns were conditioned with 1 ml of methanol, equilibrated with 1 ml of 100 mM KH_2_PO_4_, the sample was loaded and washed, under vacuum, first with 1 ml of 100 mM KH_2_PO_4_ and then with 1 ml of methanol, the column was left to dry for 5 min under full vacuum, and thereafter eluted with 3 ml of ammonium hydroxide: methanol (5∶95). The resultant extract was evaporated until dry in a GeneVac rotary evaporator and stored until analysis.

### Leaf and flower alkaloid analysis

The dried leaf and flower samples were dissolved in 500 µl of 99% HPLC grade Chromasolv dichloromethane (DCM) and left to stand for an hour. A 10 µl aliquot was transferred to a clean vial and evaporated under a gentle stream of charcoal scrubbed nitrogen. The residue was reconstituted in 10 µl of methanol to which 10 µl of internal standard *n*-heptadecane (*c*. 3 mg in 4 ml of DCM) was added.

Gas chromatography (GC) analysis was carried out on a HP 5890 series II gas chromatograph (Büblingen, Germany) equipped with a flame ionisation detector (FID) and a HP-5MS column (25 m×0.20 mm ID×0.33 µm film thickness). Helium was used as a carrier gas with a column pressure of 26.3 psi and injection temperature of 250°C. One µl of the sample was injected in the split-less mode, with the oven temperature programmed at 60°C for 0 min, ramped at the rate of 8°C/min to 200°C, held for 10 min, then ramped at 10°C/min to 300°C.

Chromatograms were recorded and the peak areas quantified using HP Chemstation software. Nicotine, nornicotine and anabasine were identified based on comparison with retention times of synthetic standards. Quantification was achieved by comparing the relative mass ratios (RMR) of each of these compounds in a standard solution mixture (*c*. 1 mg nicotine, *c*. 0.3 mg nornicotine and *c*. 0.4 mg anabasine in 4 ml DCM) relative to the RMR of *n-*heptadecane. The standard mixture was run before each batch of samples in order to account for any variability. All reagents were of analytical grade and purchased from Sigma Aldrich (Munich, Germany).

### Nectar alkaloid analysis

Deionised water (200 µl) was added to each nectar sample. The samples were then sonicated for about 5 min until they had re-dissolved and thereafter centrifuged at 10,000 rpm to remove any insoluble components. Each aqueous nectar sample was partitioned with 200 µl chloroform and mixed on a whirlimixer for one minute. The chloroform partition was allowed to settle and 2 µl were injected directly onto a DB5 non-polar column (30 m×0.25 mm i.d. ×0.25 µm film thickness) housed in a GC-MS 6890N GC (Agilent), linked to a 5973 MSD (Agilent). The carrier gas was helium at 1 ml/min and oven temperature was held at 60°C for 2 min, and programmed at 6°C/min to 250°C.

As a control, we also collected the nectar of *N. benthamiana* plants (*n* = 20), a species known to contain alkaloids in the nectar [31], for nicotine analysis. Flowers of *N. benthamiana* are much smaller than those of *N. africana*, thus to collect the nectar we gently pulled the flowers from the peduncle and squeezed the corolla so that nectar emerged from the flower base, which was then collected with a 10 µl glass microcapillary tube. The nectar was pooled across plants so that at least 20 µl were collected per sample. Nectar samples were partitioned and analysed as described above for *N. africana* nectar.

### Statistical analysis

Data were tested for normality and homogeneity of variances. The relative growth rate of larvae was analyzed with a generalized linear model (GLM) [44] with a gamma distribution for errors and a log link function. Means were separated using *t* probabilities of pairwise differences. The effect of larval density on the leaf area consumed was analyzed by analysis of variance (ANOVA). Means were separated using Fisher’s protected least significant difference (LSD) test. Generalized linear modelling using a log link function with the gamma distribution was used to test for differences in nicotine, nornicotine and anabasine concentrations in top, middle and bottom leaves, as well as the effect of different herbivore densities on the concentration of each alkaloid in a) damaged and undamaged middle leaves and b) undamaged top and undamaged bottom leaves of herbivore damaged compared to undamaged plants. Means were separated using *t* probabilities of pairwise differences. The significance level was set at *P*<0.05 for all analyses. Data were analyzed with GenStat [45] and SPSS (IBM SPSS Statistics, version 21).

The sole alkaloid recorded in flowers (four out of seven samples) was nornicotine, and this at very low concentrations only. The nectar of *N. africana* contained no alkaloids at all. Therefore no statistical analyses were performed on the flower and nectar samples.

## Results

Plants did not become pot-bound during the study – we examined this by removing plants from pots after the experiment, and checked that the roots had not taken on the shape of the pot.

**Figure 2:**
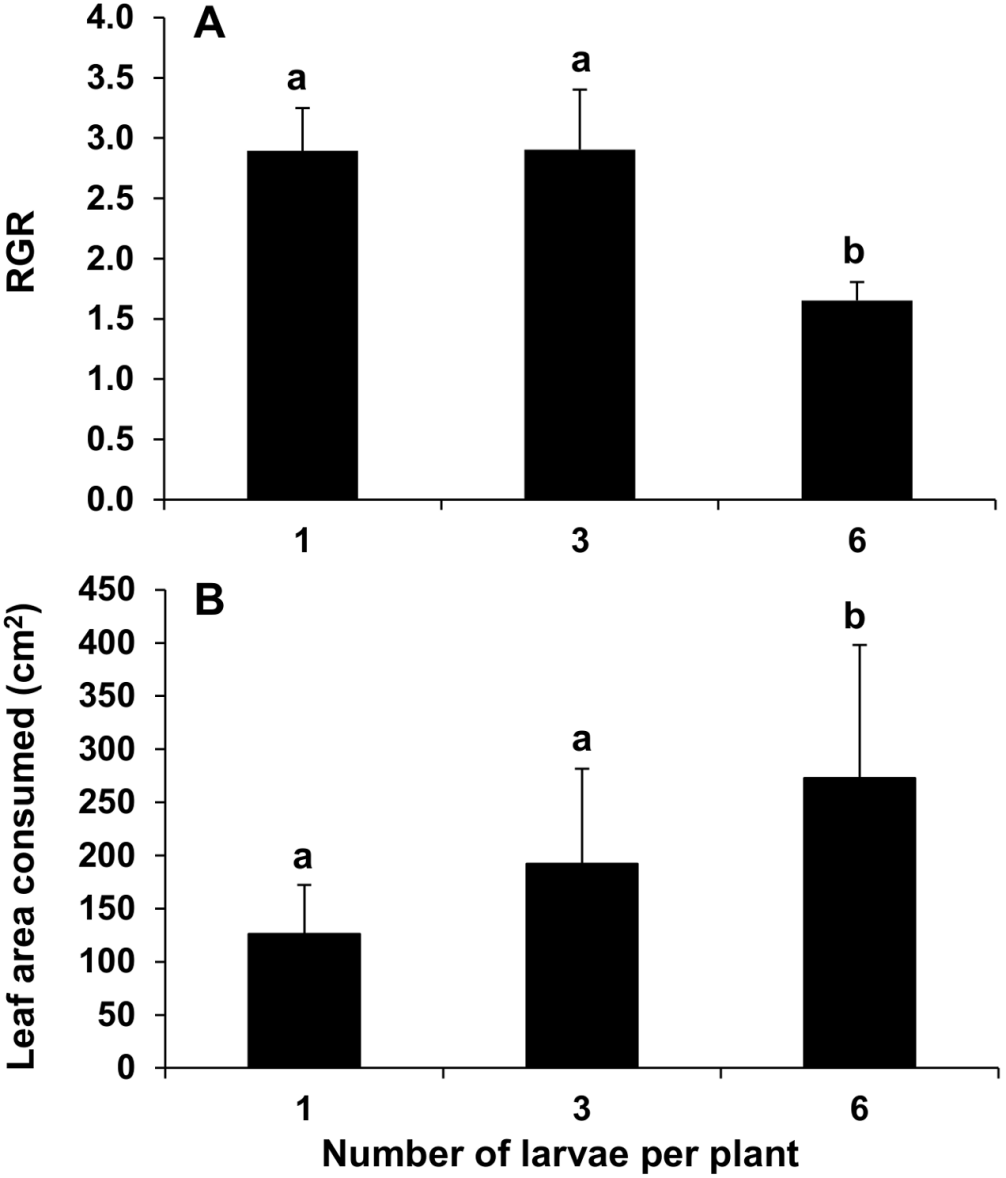
Larval growth and consumption. (A) The relative growth rate (RGR) of fourth-instar *Helicoverpa armigera* larvae feeding on flowering *Nicotiana africana* plants for four days and (B) the leaf area consumed per larva (mean ± SE). One (*n* = 5), three (*n* = 15) or six larvae (*n* = 30) were exposed to single leaves per plant. Same letters above bars indicate no significant differences at *P*>0.05.

When plants were exposed to six larvae, the relative growth rate of larvae was significantly reduced after the four-day feeding period compared to those larvae feeding on plants exposed to one or three larvae (deviance ratio = 6.47, *df* = 2, *P*<0.003; Figure 2a). Relative larval growth was reduced 1.8-fold on plants exposed to six larvae, although leaf area consumption per larva was significantly higher at double the amount consumed by larvae feeding singly on *N. africana* plants (*F*_2,45_ = 4.68, *P* = 0.014; Figure 2b).

The constitutive alkaloid content of the leaves (µg/g dry mass, mean ± SE) of control plants was as follows: nicotine 0.66±0.21, nornicotine 28.03±12.66 and anabasine 4.95±3.86). The nornicotine concentration was significantly higher than the nicotine or anabasine concentrations (Wald = 49.02, *df* = 2, *P*<0.0001). Nornicotine made up approximately 83% of the alkaloid content, followed by 15% anabasine and 2% nicotine. The top leaves of control plants (29.06±13.95) had a significantly higher total alkaloid concentration than middle (2.15±1.16) or bottom (4.66±3.43) leaves (Wald = 26.47, *df* = 2, *P*<0.0001). There was no significant alkaloid x leaf position interaction effect (Wald = 2.87, *df* = 4, *P = *0.580).

**Figure 3:**
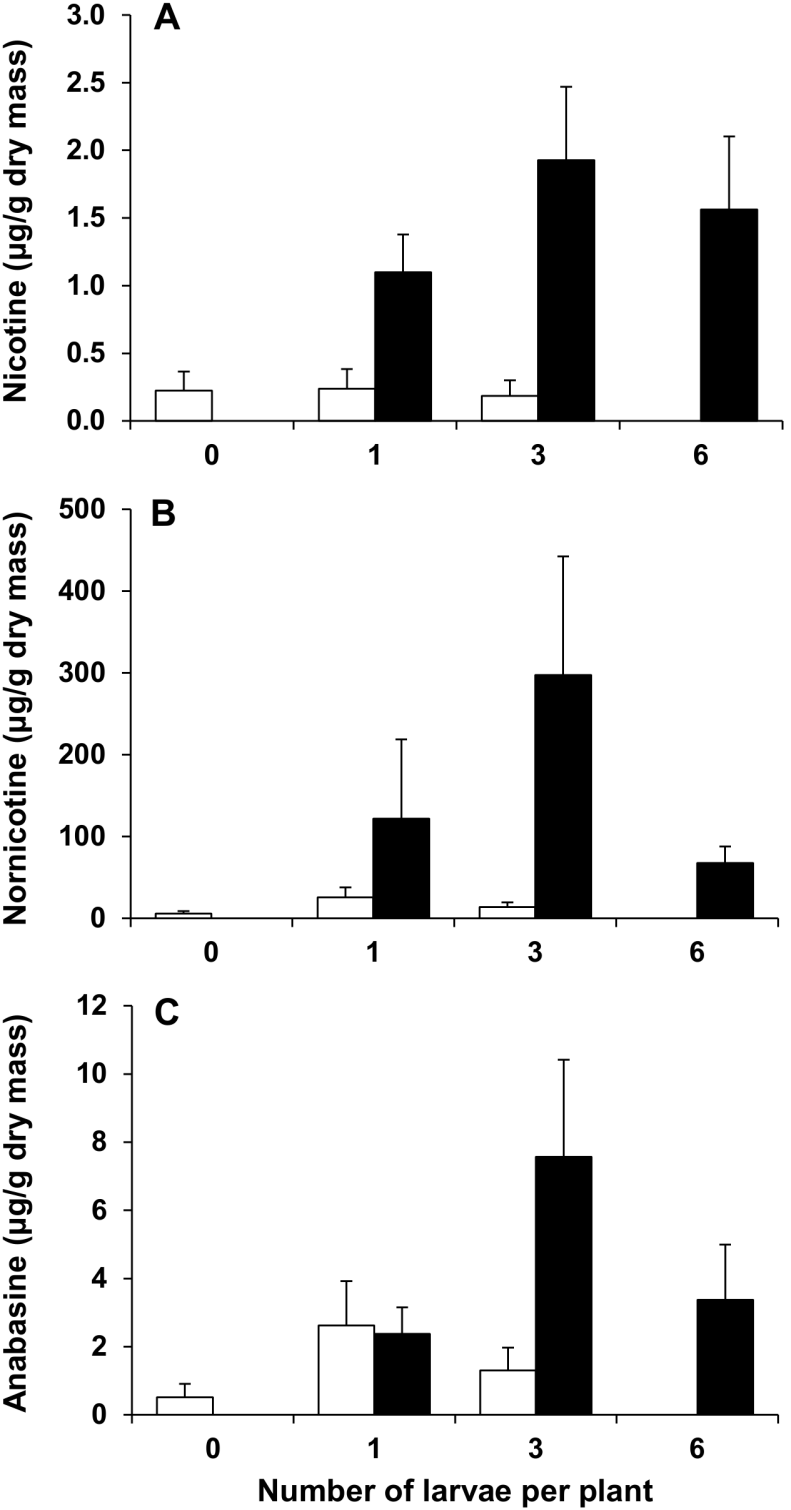
Effect of herbivory on leaf alkaloid concentrations. Effect of herbivory by *Helicoverpa armigera* fourth-instar larvae on (A) nicotine, (B) nornicotine and (C) anabasine concentrations (mean ± SE) in undamaged (open bars) and herbivore damaged (black bars) middle leaves of flowering *Nicotiana africana* plants. *n* = 4–30.

Plants damaged by herbivory had significantly higher nicotine and nornicotine concentrations in herbivore-damaged (one and three larvae) compared to undamaged middle leaves (Table 1, Figure 3a, b). Nicotine concentrations increased 5-fold (one larva) to 10-fold (three larvae) and nornicotine concentrations 5-fold (one larva) to 22-fold (three larvae). Neither larval density (one and three larvae) nor the interaction between the two effects were significant. There was no significant difference in the anabasine concentrations between damaged and undamaged middle leaves nor between different larval densities nor was there any interaction between the two effects (Table 1, Figure 3c). Leaves from control plants and plants exposed to six larvae were not included in the analysis of middle leaves because no damaged and undamaged leaves, respectively, were available (Figure 3). The nicotine and anabasine concentrations in damaged middle leaves exposed to one, three and six larvae did not differ significantly (nicotine: Wald = 0.53, *df* = 2, *P* = 0.766; anabasine: Wald = 2.85, *df* = 2, *P* = 0.241). The nornicotine concentration in damaged leaves of plants exposed to three larvae was significantly higher than in those exposed to six larvae (Wald = 7.71, *df* = 2, *P* = 0.021). Differences between damaged leaves from plants exposed to one and three larvae, and one and six larvae, were not significant.

**Table 1:** Results of generalized linear model (GLM) analyses for alkaloid concentrations in *Nicotiana africana*.

	Nicotine			Nornicotine			Anabasine		
Effect	Deviance ratio	*df*	*P*	Deviance ratio	*df*	*P*	Deviance ratio	*df*	*P*
**Damaged vs undamaged middle leaves**									
Treatment (damage vs undamaged)	7.11	1	**0.013**	5.88	1	**0.023**	1.32	1	0.261
Larval density (1 and 3 larvae)^1^	1.72	1	0.202	1.96	1	0.173	1.37	1	0.253
Treatment x larval density	0.37	1	0.551	0.79	1	0.383	1.44	1	0.241
**Undamaged top vs undamaged bottom leaves**									
Leaf (top vs bottom)	19.59	1	**<0.001**	50.39	1	**<0.001**	18.04	1	**<0.001**
Larval density (0, 1, 3 and 6 larvae)	0.33	3	0.804	0.55	3	0.651	2.04	3	0.126
Leaf x larval density	2.69	3	0.062	3.13	3	**0.038**	1.29	3	0.293

1 Data for undamaged leaves from control plants as well as damaged leaves from plants with six larvae were not included in the analysis because no damaged and undamaged middle leaves, respectively, were available.

**Figure 4:**
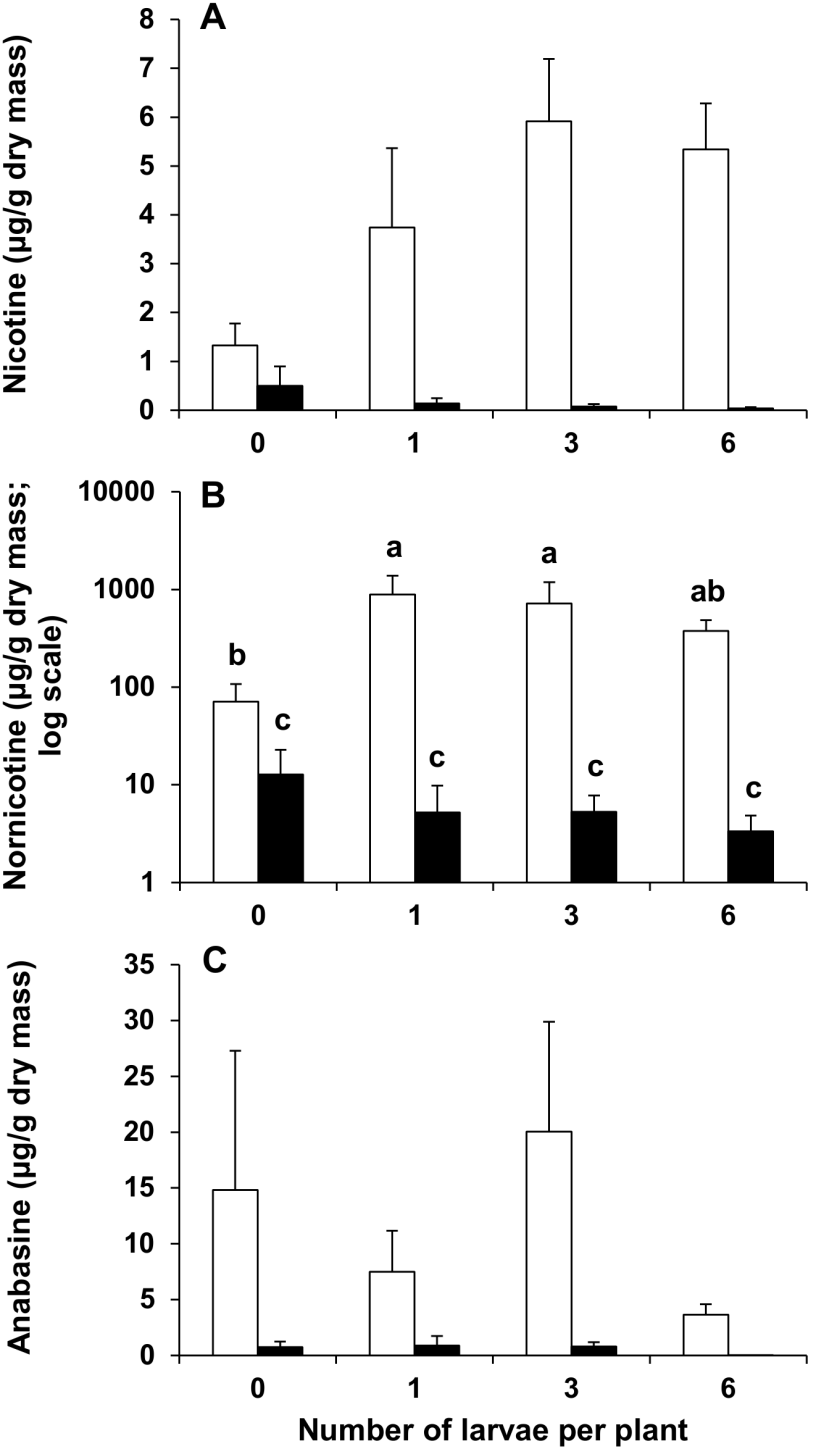
Alkaloid concentrations in undamaged leaves after herbivory. Effect of herbivory by *Helicoverpa armigera* fourth-instar larvae on (A) nicotine, (B) nornicotine and (C) anabasine concentrations (mean ± SE) in undamaged top (open bars) and undamaged bottom (black bars) leaves of flowering *Nicotiana africana*. *n* = 3–8. Same letters above bars indicate no significant differences at *P*>0.05.

Top leaves had significantly higher nicotine concentrations than bottom leaves (Table 1). Larval density did not influence nicotine concentrations and there was no significant leaf position x larval density effect (Table 1, Figure 4a). The nornicotine concentration was significantly higher in top leaves compared to bottom leaves. Larval density had no effect on nornicotine concentrations but the interaction between leaf position and larval density was significant (Table 1). Although the mean concentration of nornicotine was higher in top leaves of herbivore damaged plants compared to top leaves of undamaged plants and bottom leaves, differences were significant only for top leaves of plants exposed to one and three larvae. The nornicotine concentration in top leaves increased 10-fold (three larvae) to 13-fold (one larva) (Fig. 4b). Anabasine concentrations were higher in top compared to bottom leaves. There was no significant difference in anabasine concentration between different larval densities, or in the interaction between leaf position and larval densities (Table 1, Figure 4c).

The only alkaloid recorded in the flower samples was nornicotine, and this in low concentrations in only four out of the seven samples analyzed (2.09±0.79 µg/g dry mass, mean ± SE). *Nicotiana africana* nectar did not contain any of the three alkaloids. Nectar of *N. benthamiana*, analyzed in the same way as that of *N. africana*, contained 19.1±2.3 ng/µl nicotine (*n* = 9 samples, pooled across 20 plants) and the amount was similar to that recorded earlier by Adler et al. [31].

## Discussion

Of the three leaf alkaloids of *N. africana* analyzed, nornicotine made up approximately 83% of the total, followed by 15% anabasine and 2% nicotine. That nornicotine is the principal alkaloid of *N. africana* is in keeping with the findings of Saitoh et al. [19] and Sisson and Severson [38]. The concentration of the fourth main alkaloid in *Nicotiana* species, anatabine [18], was not determined in the current study but constituted between 3 and 7% compared to 0.3 and 0.8% anabasine of the total alkaloids measured in leaves of *N. africana* by Saitoh et al. [19] and Sisson and Severson [38].

The constitutive alkaloid contents of *Nicotiana* species vary considerably in concentration and composition [19]. For example, Saitoh et al. [19] recorded *c*. 6260 µg/g of leaf nornicotine in *N. africana* compared to 28 µg/g in our study. The discrepancies between studies may be due to differences in the growing environment. For example, Sisson and Severson [38] found that total alkaloid levels in *Nicotiana* plants grown in a greenhouse differed nearly 200-fold and in field-grown plants 400-fold. The authors suggested that the type and amount of alkaloid produced may be influenced by factors such as different culture conditions and seed sources. Light intensity, nutrients and water supply have been shown to influence the alkaloid content in tropical trees [46] and in *Nicotiana* species [17], [27].

Herbivory increased nornicotine levels in the leaves of *N. africana* both systemically and at a localized leaf level. The nornicotine concentrations in undamaged top leaves of herbivore-damaged plants (one and three larvae) increased compared to top leaves of undamaged plants. This is similar to the North American *N. repanda* and *N. trigonophylla*, two nornicotine accumulating species, where nornicotine concentrations increased in undamaged leaves of plants with mechanically damaged leaves [16]. Likewise, *N. attenuata* and *N. sylvestris,* which have nicotine as their principal alkaloid, show an increase in the production of the alkaloid when attacked by herbivores or damaged mechanically [16], [47]. The increase of nornicotine in undamaged young leaves of *N. africana* is consistent with the optimal defense theory [9]. In keeping with this theory, younger (top) leaves had higher alkaloid concentrations than middle or bottom leaves in flowering *N. africana*. Similarly, the allocation of nicotine in *N. sylvestris* was higher in young than mature or old leaves, and younger leaves of flowering plants were better defended than older leaves [26].

Both nicotine and nornicotine concentrations increased in attacked leaves in flowering *N. africana* plants exposed to *H. armigera* larvae. This suggests that nicotine, as well as nornicotine, is important as an induced defense in *N. africana* plants. There is a lack of information on whether nicotine, when not the main alkaloid, increases in other *Nicotiana* species when attacked. However, the synthesis of nornicotine is secondary; it is the demethylation product of nicotine [18]. Nicotine is synthesized in the roots and transported to the leaves whereas nornicotine is produced in the roots and leaves [48]. Possible reasons for nicotine accumulation in leaves in response to herbivory in *N. africana*, and we stress that these are speculative, could be that nicotine was not yet converted to nornicotine, or that the production of nicotine as defense in nornicotine-dominated plants may lower the cost of defending the plant against herbivory, due to saving on nornicotine biosynthesis costs.

Differences in low (one individual) and medium (three individuals) larval density had little influence on alkaloid production in our study. However, larval growth was reduced and leaf area consumption per larva increased when six *H. armigera* larvae fed on a plant compared to one and three larvae. This suggests that alkaloid concentration in damaged leaves increased with the severity of damage. However, the nornicotine concentration in damaged leaves of plants with six larvae was lower than that of plants exposed to three larvae. This could be because the cost of producing alkaloids in a plant that is already under severe herbivore attack (six larvae) compared to mild to moderate attack (one or three larvae) may outweigh the benefits, and the plant may rather invest in new growth, i.e. grow new leaves as opposed to protecting older and already attacked leaves e.g. [12], [49]. A mixed defense strategy could involve both tolerance (increased growth) and resistance (production of alkaloids) [2], [3], [14], with tolerance and resistance each having associated costs and benefits that may differ within populations [14].

We expected the nectar of *N. africana* to contain alkaloids, but our results showed otherwise and this is not unusual; a number of *Nicotiana* species, e.g. *N. alata* and *N. bonariensis,* do not contain alkaloids in their nectar [31]. Kessler et al. [50] showed that pollinators removed more nectar from *N. attenuata* plants in which a nicotine-producing gene was silenced than from plants where the nectar contained nicotine. The alkaloid-free nectar of *N. africana* may be an advantage in that the heightened expression of alkaloids in the leaves and other plant parts does not affect the pollination success of the species. Marlin et al. (unpublished data) found that *N. africana* can reproduce through self-pollination, but seed and fruit set increased when the plants were cross-pollinated, and sunbirds have been suggested as potential pollinators of this species (Marlin et al. unpublished data) [51]. However, nicotine in nectar does not necessarily deter pollinators; sunbirds are deterred by nicotine only when the concentrations are higher than 3 µM [52], [53], and Manson et al. [54] found that natural concentrations of alkaloids in the nectar of the montane herb *Delphinium barbeyi* did not deter bumblebee pollinators. This is likely because bumble bees have poor taste acuity for nectar toxins, including nicotine, such that they are not able to detect them at natural concentrations but only when the toxins increase above natural concentrations [55]. The floral tissue of *N. africana* contained very low levels of nornicotine. This is the only study of which we are aware in which the nornicotine content of flowers has been quantified in a *Nicotiana* species. Adler et al. [31] found that many *Nicotiana* species contained mostly nicotine but not anabasine in the flowers, and this was reflected in their leaves which similarly contained more nicotine than anabasine. However, these authors did not measure nornicotine in any of the plant tissues. *Nicotiana africana* flowers contain nornicotine only and this is reflected in the leaves which contain more nornicotine than nicotine or anabasine.

Attempts to grow *N. africana* plants in the field in the Pretoria region (Gauteng, South Africa) failed and plants are difficult to access in their native range. The three populations of *N. africana* occur in disjunct and isolated patches along the edge of the Namib Desert [56]. However, alkaloid responses to herbivore damage in *N. africana* need to be demonstrated in the field to validate our results, because alkaloid induction in greenhouse studies does not necessarily concur with that of field-grown plants [16].

In summary, whether a plant expresses herbivore-induced defenses depends on a number of factors such as the plant species, the amount and the type of damage inflicted, the species of herbivore inflicting damage as well as the feeding guild of the herbivore. Whilst there are no field observations on *N. africana*, it is unlikely that the species does not come into contact with any enemies. Although the constitutive leaf alkaloid levels of *N. africana* would deter herbivores, herbivory does induce alkaloid production in this species, offering further defense should herbivory occur. The combination of high leaf alkaloid levels, which deter herbivores, but alkaloid-free nectar, which does not deter pollinators, may be an adaptive trait in a species such as *N. africana*. These plants grow at the edge of the Namib Desert in isolated habitats that are rarely exposed to pollinators, but are among few food sources available to herbivores in this semi-arid environment: there would be an ecological advantage if induced defenses did not compromise the pollinator reward.
